# Fast robot-to-iOCT calibration using conical geometry and 2D cross-scan OCT

**DOI:** 10.1117/1.JBO.31.12.123305

**Published:** 2026-08-27

**Authors:** Krzysztof Gromada, Tomasz Piesio, Piotr Kasprzycki, Andrea Curatolo, Karol Karnowski

**Affiliations:** a International Centre for Translational Eye Research, Warsaw, Poland; b Polish Academy of Sciences, Institute of Physical Chemistry, Warsaw, Poland; c Politecnico di Milano, Department of Physics, Milan, Italy; d The University of Western Australia, Department of Electrical, Electronic and Computer Engineering, Perth, Australia

**Keywords:** optical coherence tomography, computer vision, robot-assisted surgery, coordinate system calibration.

## Abstract

**Significance:**

Accurate spatial registration between optical coherence tomography (OCT) and robotic coordinate systems is critical for OCT-guided microsurgery, where precise tool localization is essential for safe, reliable intervention.

**Aim:**

We propose and validate a practical and accurate method for calibrating the transformation between OCT and robot coordinate systems in robotic microsurgical systems.

**Approach:**

A conical calibration tool rigidly attached to the robot wrist was scanned using fast 2D OCT cross-sections. After denoising, edge extraction, and two-line fitting, the cone tip was localized, and the resulting coordinates were incorporated into a vector-based three-point calibration to recover the OCT-to-robot transformation. The method supports both manual and automated operation, including pose adjustment to improve scan-plane alignment.

**Results:**

Experiments with a swept-source OCT system, a Meca500 robot, and a 3D-printed calibration tool showed stable registration across the OCT working range. The method also remained robust in the presence of OCT noise and moderate manufacturing imperfections of the cone geometry. The single cone-tip localization repeatability was estimated to be 34.7  μm, where calibrating over 16 mm of OCT X-Y lateral field-of-view range translates to 0.31% of linear calibration uncertainty in a bench-top environment.

**Conclusions:**

The proposed cone-based framework provides a practical and accurate solution for routine robot-to-OCT calibration in high-precision surgical systems.

## 1 Introduction

Robot-assisted surgeries have emerged as a groundbreaking advancement in medical technology, revolutionizing the performance of complex procedures.^1^ This is particularly significant in eye surgery, especially when combined with intraoperative optical coherence tomography (iOCT) guidance.^2^ The combination of a robotic system’s precision and OCT imaging’s accuracy improves the likelihood of successful surgery, enhances patient safety, and reduces healing times.^3^

Robot-assisted surgeries leverage the dexterity and stability of robotic arms, allowing surgeons to perform intricate maneuvers with greater precision and reduced tissue trauma.^1^^,^^4^ Incorporating intraoperative OCT (iOCT) guidance further elevates surgical capabilities by providing high-resolution, real-time imaging of internal anatomical structures.^5^ This detailed visualization allows surgeons to navigate delicate tissues and organs with enhanced accuracy, minimizing the risk of inadvertent damage and complications.^6^

In ophthalmic surgery, iOCT guidance has significantly transformed procedures,^7^ such as corneal surgeries,^8^ including partial corneal transplants, and vitreoretinal surgeries,^9^ including epiretinal or inner limiting membrane peeling and delivery of therapeutic agents, like gene therapy, to the subretinal space.^10^ Surgeons can precisely assess ocular tissue condition and identify subtle abnormalities, leading to more targeted interventions and improved postoperative outcomes.^11^ In addition, the minimally invasive nature of robotic-assisted surgeries could reduce ocular trauma and discomfort,^12^ accelerates recovery times, and decreases hospital stays, leading to cost savings and improved patient satisfaction. The combination of iOCT guidance with robotic-assisted microsurgery is showing extensive potential in ophthalmic procedures, such as deep anterior lamellar keratoplasty,^13^^,^^14^ semi-automated cataract removal,^15^ retinal vein cannulation for retinal vein occlusion,^16^ and subretinal injections.^17^

However, despite these advantages, challenges such as high initial costs, limited accessibility, and ongoing technical advancements remain. One key issue in these systems is the calibration of multiple coordinate systems.^18^

### 1.1 Main Coordinate Frames in the System

iOCT-guided robotic microsurgeries involve a complex interplay among multiple coordinate systems to ensure accurate imaging, surgical guidance, and seamless collaboration among the surgeon, robot, and imaging devices. Proper understanding and integration of these coordinate systems are essential to achieving precise and successful surgical outcomes.

**Fig. 1:**
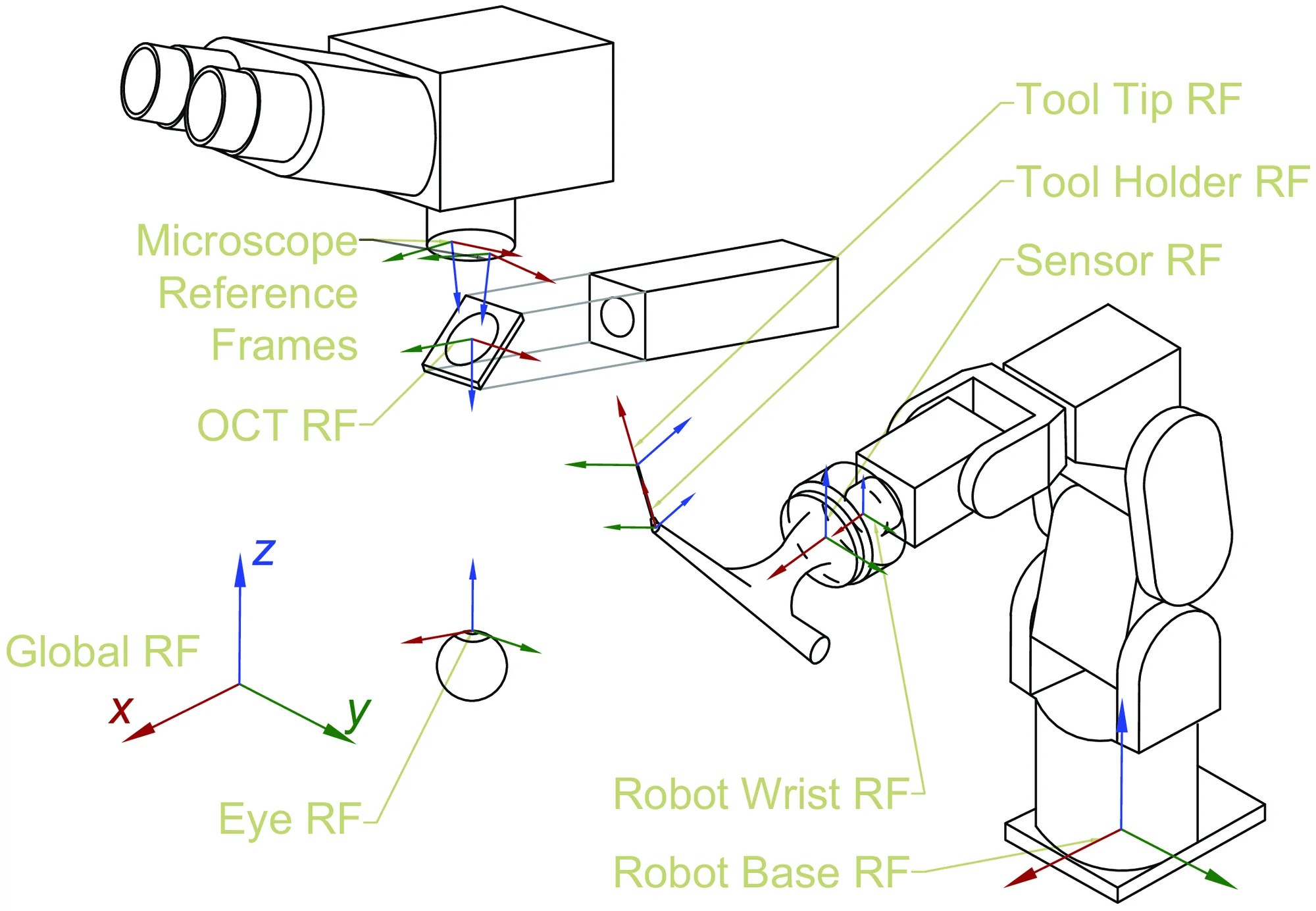
Reference frames for the hardware in a robot-coupled OCT system.

To better understand the different coordinate systems involved in iOCT-guided robotic microsurgery, a visualization is presented in Fig. 1, where we shall focus on calibration relevant to ocular anterior segment procedures:

1. Microscope reference systems—the microscopes typically have skewed projections for the eyes to provide stereoscopic depth information to the user. In microscope-integrated iOCT (MIOCT) systems, two cameras are usually mounted on the surgical microscope to record corresponding eye images. We can assume that the cameras see the same image +and share the same reference systems as the eyes. As such, two reference systems are needed to integrate microscope readouts with the system. The proposed definition defines the Z axis as the depth of each projection, whereas X and Y correspond to the camera’s pixel orientation.
2. OCT reference system—the MIOCT system is installed below the body of the surgical microscope and just above the microscope objective lens (not depicted in Fig. 1). The beam generated by the system is reflected off a set of two galvanometric mirrors that enable scanning the beam at the sample plane with arbitrary patterns, in the case of the described system, a crosshair scan.^19^^,^^20^ As such, another reference system is introduced. The Z axis is located on the microscope’s optical symmetry line, whereas Y and X should correspond to the microscope’s coordinate system. This reference system allows precise alignment of the scanning area with the surgical field.
3. Robot reference system—the robot used in robot-assisted surgeries has its own global reference system, often located at the robot’s base. This reference system provides a fixed point of origin and orientation for the robot’s movements. Each robot joint also has its individual reference system, which we will not describe in detail here. At the robot’s end effector, additional reference systems are crucial for surgical guidance and control. The first reference system is located at the robot’s wrist, enabling precise orientation adjustments. Furthermore, the robot may employ force and torque sensors,^21^ which introduce another coordinate system at the end of the sensor. These sensors enable compliance control of the robot during robot-assisted surgery.
4. Tool reference systems—two additional and crucial reference systems are attached to the robot wrist/sensor. They correspond to the toolholder reference and the final tool tip. The toolholder reference system can be treated as constant in reference to the robot wrist and sensor. Tool tip position, on the other hand, can change during surgery due to the tool change and the tool’s limited stiffness.
5. Patient and eye reference systems—two reference systems can be defined for the patient/global coordinate system and the eye. As the eye can move during surgery due to involuntary eye movements or surgeon-induced adjustments, a separate eye-specific coordinate system should be used in the analysis. The eye coordinate system helps track and compensate for any shifts or movements in the eye’s position during the procedure, maintaining accurate OCT imaging and surgical planning.

Typically, the iOCT system is coupled to the microscope, meaning the position of the OCT reference system is fixed relative to both microscope reference systems. As mentioned before, the same occurs for the robot reference systems and the tool holder. In the classic approach, all other coordinate systems are free to move relative to each other.

To enable the system to function at a minimum, the OCT reference system must be calibrated using information about the tool tip position. Classically, in iOCT-guided surgeries,^22^^–^^24^ tool tip tracking is performed using microscope image processing, which requires calibration of the OCT relative to the microscope. Alternatively, in robot-assisted surgery, the tool tip position can be fed by the robot arm controller, which is a more robust solution, provided a rigid mount of the OCT and robot and assuming minor tool deflections and image refraction inside the tissue of interest.

Our solution addresses robot arm-to-OCT (or robot wrist-to-OCT) calibration. Such calibration is tool-independent and requires a one-time calibration for an iOCT setup, provided that the robot arm is rigidly mounted to the OCT. This calibration aims to deliver information about the additional reference frames and does not directly translate to tool tracking. It can be indirectly used for tool tracking or additional data acquisition. The technical considerations for its implementation, along with the resulting data that can be obtained, are described in detail in Sec. 5.

Both the eye and patient/world reference systems can move with respect to the other systems. For that reason, their use is practical only in automated surgeries, where targets are defined in the eye reference system, and their position must be tracked during the surgery.

## 2 State of the Art

The broader problem of determining the spatial relationship between a robot and a sensor is classically formulated as a hand–eye calibration. There are two types of hand–eye coordination: eye-in-hand, where the sensor is connected to the robot arm (typically end effector), or eye-to-hand where sensor observes the robot from a stationary position. Classically, the problem is represented as a AX=XB matrix equation, where X is the unknown sensor-to-robot transformation, A and B represent the poses of the robot end-effector and the sensor. The matrices are estimated from multiple measurements acquired at different robot poses, after which X is determined using optimization or closed-form calibration methods.

Multiple algebraic solutions were established over years for different eye-in-hand configurations by Tsai and Lenz,^25^ Park and Martin,^26^ and others.^27^^,^^28^ Some recent articles expand the possibilities of quick eye-to-hand calibration.^29^^,^^30^ State-of-the-art hand–eye calibration methods require the sensor to produce a full 6-degree-of-freedom pose (rotation and translation) for a calibration target at each robot configuration. Usually, multiple poses of a precisely defined calibration tool have to be measured to account for camera distortions enabling 6D pose estimation of a calibration pattern. The iOCT system used here, however, does not produce a pose for the calibration geometry. It produces only a 3D point location derived from the intensity cross-section of the conical tool. Applying AX=XB solvers directly is therefore not straightforward without first solving the point localization problem that the present method addresses. The following solution and all cited calibration algorithms are by definition hand–eye calibration problem, usually eye-to-hand due to OCT size requirements. Our pipeline can be viewed as providing the sensor-side primitive that enables AX=XB-based solvers to be applied in future extensions requiring multipose calibration.

The task of robot and OCT system calibration is presented in an article by Yu et al.^22^ The authors built a specialized, parallel-kinematics robot for microvascular retinal surgery, which required kinematic analysis and calibration. Separately, the OCT probe is calibrated, by scanning the tooth pattern of a precision-grade M3×0.5 metric screw. This enables the definition of the OCT scan resolution using two methods, which exhibit a considerable 3.4% deviation in the best-case scenario. The authors focus only on pixel-resolution calibration while also noting the need to implement a nonlinear calibration algorithm. Due to lateral distortion, the pixel resolution depends on the position of the element in the OCT scan. In addition, the entire calibration procedure is conducted manually and does not provide a method for accurately positioning the calibration tool, which can be skewed. To fix both of these problems, the calibration has to be automated (to enable multiple scanning readouts over the OCT Field of View), and a new calibration geometry, which limits or removes the skewing influence, has to be introduced.

A different problem was solved by Zhou et al.,^18^ who proposed a calibration algorithm between the needle tip (considered part of the robot) and the OCT system. The proposed calibration algorithm is based on a volumetric OCT scan, which enables cloud-point-based geometry fitting. The tool must be moved in 15 steps along all three axes, by 20  μm each time. The measurements were compared with a classic calibration algorithm using a specialized tool with a ball marker on top. The resulting error values were under 10  μm. The authors do not define the details of the coordinate system calibration but rather focus only on tool tip position estimation. In robotics, multiple approaches enable coordinate system calibration using single-point localization. One of the most popular is three-point calibration, which is widely used and has been extended in the literature,^31^^–^^33^ especially when calibrated using multiple-camera systems.^34^^,^^35^

Mach et al. proposed an OCT-guided robotic framework for subretinal needle placement, in which a steady-hand–eye robot is registered to a 3D swept-source OCT volume using marker-free calibration.^36^ Data acquisition consisted of moving the robot-mounted needle to nine predefined positions, recording the corresponding robot coordinates, and acquiring OCT volumes at each position. A deep-learning-based 3DU-Net model automatically segmented the needle and identified the needle tip within the OCT data, providing corresponding image-space coordinates for registration. Last, OCT-to-robot transformation was estimated using a Levenberg–Marquardt optimization. Evaluation was conducted on an aluminum target board and cadaveric pig eyes. For manually identified needle tips, the framework achieved a mean Euclidean targeting error of 24.7  μm on the board and 23.3  μm in retinal tissue. Using automatic needle-tip detection, the mean Euclidean error was 29.1  μm on the target board and 23.8  μm in retinal tissue. The reported errors were below the ∼25  μm accuracy requirement for subretinal injection, demonstrating clinically relevant precision. The authors did not report the calibration time.

A related approach, this time for anterior eye robot-assisted surgery, was proposed by Tian et al.,^37^ who presented a method for calibrating the robot base frame to the OCT frame in the context of autonomous robotic micro-suturing. The calibration process estimates the transformations between the robot and the needle frame, and the 3D OCT imaging frame. This is done using an adapted iterative closest points algorithm that aligns the observed needle surface voxels in the OCT data with a CAD model of the needle, with a focus on fitting the needle tip. The authors report calibration position root mean square error (RMSE) values of 75 to 227  μm and the rotation RMSE of 0.014 to 0.090 rad. Similar tasks of robot-assisted coordinate system calibration are presented for other sensors, such as ultrasound imaging systems^38^ or 2D lasers.^39^

The present work advances this direction by achieving low error (34.7  μm at the single-point level, corresponding to 0.31% over the full 16 mm OCT X-Y lateral FOV range) and by replacing the volumetric OCT acquisition required by prior approaches with fast 2D cross-scans,^20^ reducing the total calibration time to under 30 s.

The main novelty of this work is the complete sensing pipeline that makes standard three-point coordinate-frame estimation reliably applicable in the iOCT context. This includes a novel conical calibration geometry with a 2D OCT cross-section that provides analytically unique, sub-pixel tip localization without volumetric acquisition, with an automated alignment loop that positions the cone-tip reproducibly within the scan plane. To assess system accuracy, an algorithm for angular error estimation using the OCT system’s own measurements is proposed.

The proposed solution aims at robot-OCT calibration, not tool-OCT calibration, and is scoped to the bench-top calibration problem requiring additional solutions or assumptions to translate to real-time tool tracking for iOCT surgery. Refraction correction based on real-time corneal topography and compensation for surgical tool deflection are additionally discussed in Sec. 5.

## 3 Calibration Algorithm

The presented algorithm for coordinate system calibration is based on the analysis of a known stereometric (cone) shape. To achieve high accuracy, the definition of the shape sizes has to consider the parameters of the used OCT system.

### 3.1 OCT System Parameters

**Fig. 2:**
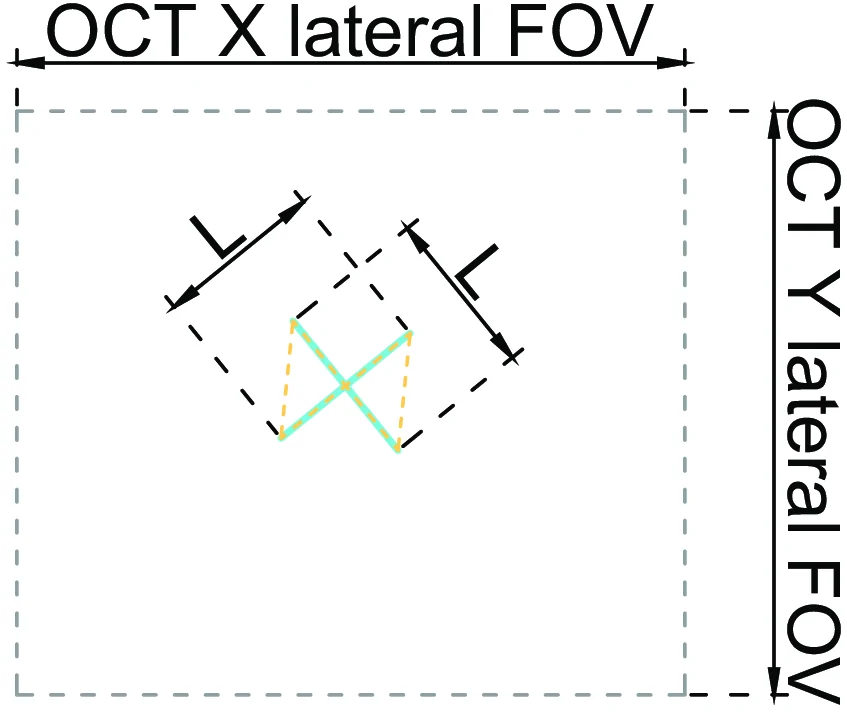
Basic dimensions of the OCT system.

In the following analysis, it is assumed that the OCT scans the area surrounding the tool tip using a cross pattern (Fig. 2). A linear scan pattern can also achieve the same results. The laser beam is controlled by mirrors attached to high-speed galvanometers. This enables high-accuracy cross-pattern scanning, as well as the movement of the pattern over larger, nonlinear areas.

The OCT range is limited by the mirror size and the galvanometer movement range (usually defined by the maximum voltage). Due to nonlinearity and dependence of the pattern orientation, simplified rectangular limits are considered. The X and Y ranges shown in Fig. 2 define a maximum-size rectangle within which the pattern’s center can move without affecting the pattern. The expected position of the pattern can be defined as a (x,y,γ) vector, where γ corresponds to Yaw—the rotation of the pattern around the Z axis. The length L defines the size of the cross pattern. It is worth mentioning that the following algorithms can also be implemented using a linear scanning pattern.

The test system presented in this work uses a swept-source OCT scheme. The light source (1060 nm MEMS-VCSEL Swept laser, Thorlabs Inc., New Jersey, United States) operates at 400,000 A-scans/second. A cross-scan is performed at 200 Hz and provides 640 A-lines in each of OCT cross-scan directions (using Saturn 1, ScannerMAX, a division of Pangolin Laser Systems, Inc., Sanford, Florida, United States).^40^ The Z-axis range (depth) of the system is $Z_{\text{range}}=6.1\;\mathrm{mm}$ in air and is a function of the laser k-clock frequency and the dual edge sampling feature of the acquisition board (Alazar Tech. ATS 9373). The X-Y scan range (L) of the OCT beam is user defined; in the described experiments, it was set to L=5.4  mm. Axial (Z axis) resolution, which is a function of the swept laser parameters (central wavelength and optical bandwidth), is 11  μm. The transverse resolution, defined by the optical elements in the sample arm, is mostly limited by the objective lens that OCT shares with the surgical microscope. The focal length of 200 mm for this lens leads to an X-Y resolution of 50  μm.

### 3.2 Calibration Geometry

OCT is efficient at measuring the geometry of an object, rather than its color, unlike cameras. For that reason, the proposed calibration pattern should be a shape that the OCT can precisely measure. Different equivalents of classic calibration patterns (e.g., a checkerboard) could be devised, but to achieve high-accuracy point estimation, a conical shape is proposed. It is worth noting that OCT systems, in general, are capable of three-dimensional geometric measurements. In the present work, however, the system operates on 2D cross-sectional scans. Restricting acquisition to 2D scans enables the proposed method to achieve very high crosshair pattern repetition speed.

**Fig. 3:**
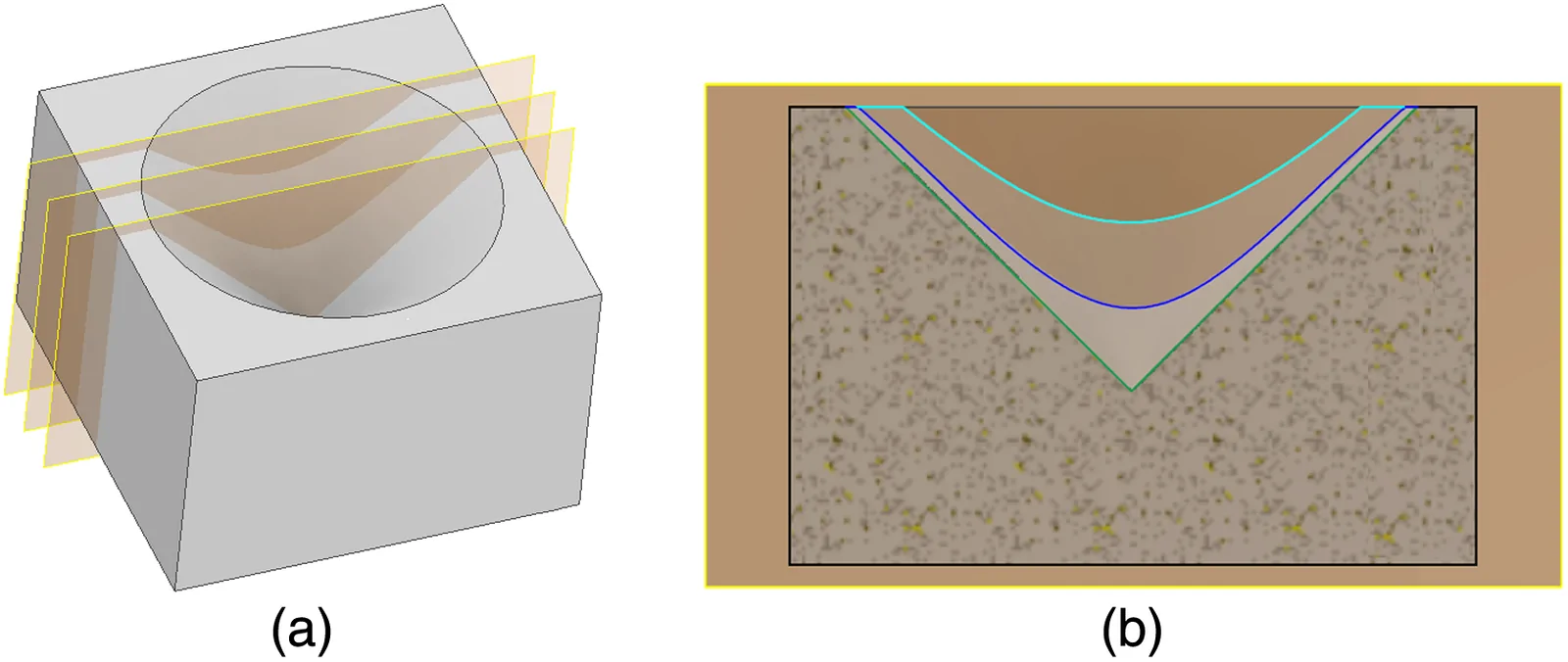
(a) 3D model of the tool used for OCT tuning with conical geometries and (b) the projections of the geometry that will be registered by the OCT during the calibration process. The hyperbolic geometries marked with three colors in panel (b) correspond to the planes in panel (a).

A simple geometry is shown in Fig. 3(a). The goal of the algorithm is to find the exact location of the cone tip, without analysis of the angular position of the tool. In addition, the scanned surfaces should cover a large area of the scan image. As such, the optimal approach is for the whole OCT pattern to fit inside the cone, so the scanned geometry spans over whole B-scan. In the geometry presented in Fig. 3(a), the cone diameter is ϕ=L+margin and height $H=Z_{\text{range}}+\text{margin}\cdot Z_{\text{range}}/L$. The proposed length margin=2  mm and value $\text{margin}\cdot Z_{\text{range}}/L$ force to keep the ratio on the cone to be similar to the scan.

The choice of a conical geometry is motivated by three properties specific to 2D OCT cross-sectional imaging:

1. The axial cross-section of a cone through its apex produces exactly two straight line segments with an intersection that is the unique cone tip; this is analytically unambiguous and straightforward to fit robustly, allowing for sub-pixel tip position estimation. By contrast, a spherical marker produces a curved arc in cross-section, requiring nonlinear fitting and making sub-pixel tip localization less reliable.
2. The rotational symmetry of the cone about its axis means that tip localization is insensitive to the azimuthal orientation of the tool, removing 1 degree of freedom that would otherwise require active control during setup. For that reason, pyramidal or checkerboard-type geometries are sub-optimal.
3. The sensitivity of the cross-sectional profile to lateral offset from the cone axis—which transitions from two straight lines at the center to increasingly curved hyperbolic arcs off center—provides a natural, continuous error signal that directly drives the automated alignment loop without any additional reference feature.

The goal of this shape is to enable precise estimation of a single point in the OCT scan. The conical shape has the advantage of different section profiles, when scanned off center [as shown in Fig. 3(b)]. A direct scan along the center line will produce two-line segments crossing at the designated point [dark green contour in Fig. 3(b)]. The further from the center, the less linear the shape is being registered (blue and turquoise section profiles). This makes the analysis of the fit easy, both manually and automatically.

The shape is easy to manufacture with any additive manufacturing tool and can also be easily created with a simple cutting operation (drilling). Both solutions are feasible as the algorithm focuses on estimating the final point based on the cone’s surface rather than detecting a single point. This increases the accuracy of point localization but also reduces the importance of cone-tip manufacturing precision. The conical shape can be either a separate calibration tool attached to the robot wrist for calibration, or a part of a surgical holder to avoid inaccuracies and time delays associated with tool replacement.

### 3.3 Coordinate System Calibration

The classic way to “teach” the robot a previously unknown coordinate system is to use a three-point approach.^31^^–^^35^^,^^41^ The simplest version assumes probing three points: first point—at the origin, second point—along the positive X axis, third point—along the positive Y axis.^41^ These three points define the direction of all the axes of the new coordinate system. The third axis is computed as the cross product of the two axes defined by the other directions. The order can vary depending on the implementation; in principle, negative axes and the Z axis can also be used. Nevertheless, this calibration requires a tool with a well-defined, measurable tip position.

In this paper, a vector-based algorithm for coordinate system estimation is proposed. A similar solution was described by Zhang et al.^32^ The original article uses an algebraic transformation to estimate the rotations between the reference systems. Its software implementation requires many edge-case checks. The following coordinate-frame reconstruction constitutes a standard final stage of the calibration procedure and operates on the OCT-localized reference points generated by the proposed pipeline. It is described in detail as it is essential to support the subsequent analysis. The proposed method determines the coordinate frame from three points measured in the robot reference frame ($P_{1}$, $P_{2}$, $P_{3}$):

• $P_{1}$ is the origin of the new coordinate system,
• $P_{2}$ is the point defining the direction of the first axis (here: the X axis),
• $P_{3}$ is the point defining the XY-half-plane (selected such that the Y coordinate is positive).

Let the displacement vectors be defined as:

$$\overset{\to }{P_{1}P_{2}}=P_{2}-P_{1},\;\overset{\to }{P_{1}P_{3}}=P_{3}-P_{1}.$$ (1)

Based on these displacement vectors, normalized axis vectors are computed:

$$\overset{\to }{V_{x}}=\frac{\overset{\to }{P_{1}P_{2}}}{\left|\overset{\to }{P_{1}P_{2}}\right|},$$ (2)

$$\vec{V_{xy}}=\frac{\vec{P_{1}P_{3}}}{\left|\vec{P_{1}P_{3}}\right|}.$$ (3)

The Z-axis direction is then obtained via cross product:

$$\overset{\to }{V_{z}}=\frac{\overset{\to }{V_{x}}\times \overset{\to }{V_{xy}}}{\left|\overset{\to }{V_{x}}\times \overset{\to }{V_{xy}}\right|},$$ (4)

and the Y-axis is defined to complete a right-handed orthogonal basis:

$$\overset{\to }{V_{y}}=\frac{\overset{\to }{V_{z}}\times \overset{\to }{V_{x}}}{\left|\overset{\to }{V_{z}}\times \overset{\to }{V_{x}}\right|}$$ (5)

The resulting rotation $R=[\overset{\to }{V_{x}},\overset{\to }{V_{y}},\overset{\to }{V_{z}}]$, together with the translation of the origin ($P_{1}$), defines the transformation matrix T:

$$T=[\begin{array}{cc} R & P_{1}\\ 0^{T} & 1 \end{array}]=[\begin{array}{cccc} \overset{\to }{V_{x_{x}}} & \overset{\to }{V_{y_{x}}} & \overset{\to }{V_{z_{x}}} & P_{1_{x}}\\ \overset{\to }{V_{x_{y}}} & \overset{\to }{V_{y_{y}}} & \overset{\to }{V_{z_{y}}} & P_{1_{y}}\\ \overset{\to }{V_{x_{z}}} & \overset{\to }{V_{y_{z}}} & \overset{\to }{V_{z_{z}}} & P_{1_{z}}\\ 0 & 0 & 0 & 1 \end{array}].$$ (6)

The transformation matrix T can be used directly for coordinate system transformation or to estimate the corresponding Euler angles of the transformation. In the described implementation, the Eigen C++ library was used. The proposed method requires more computations than the analytical approach; however, this is acceptable for calibration, and in practice, the resulting accuracy is not limited by floating-point precision.

### 3.4 Algorithm Overview

**Fig. 4:**
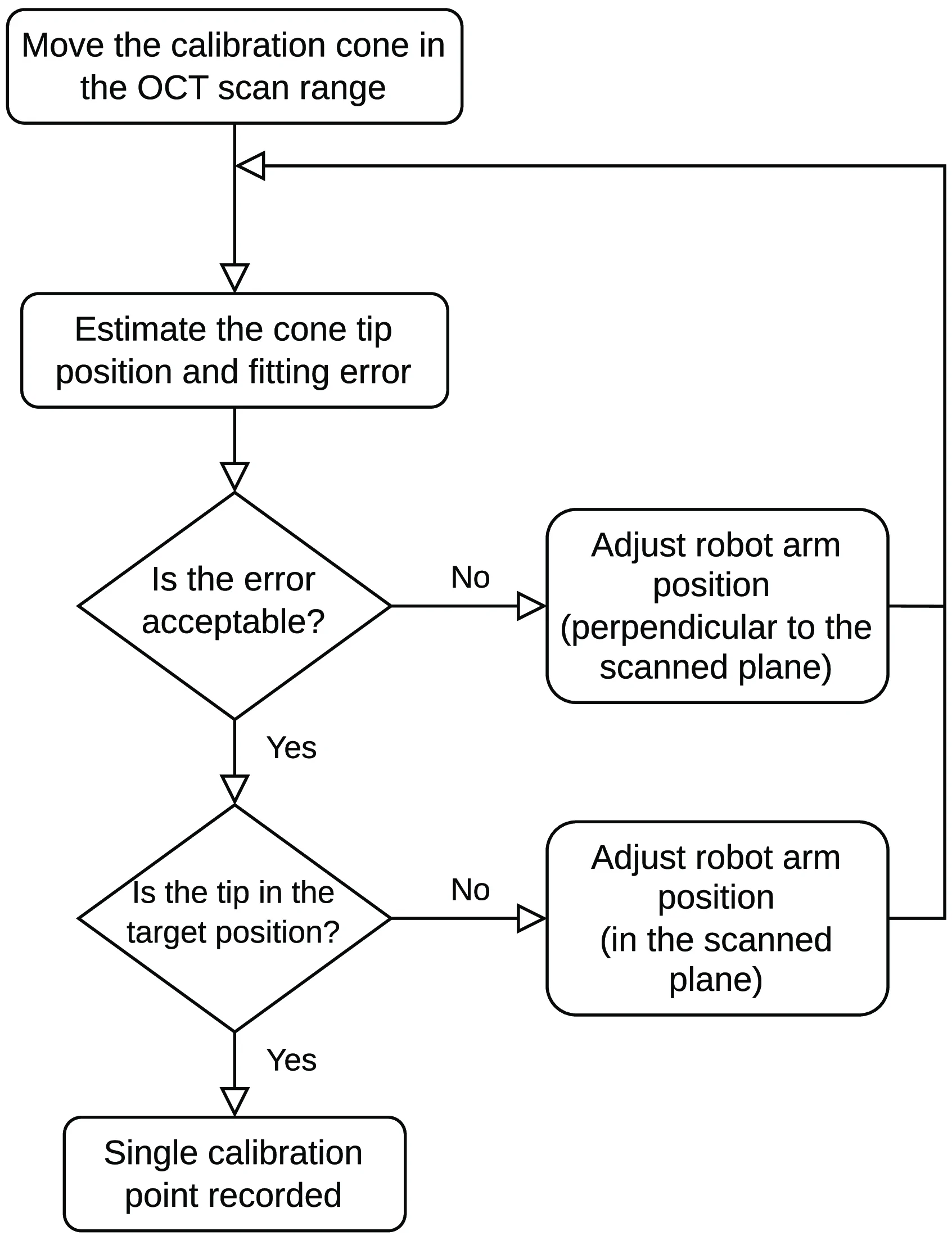
Coordinate systems tuning algorithm.

The main steps of the algorithm are illustrated in Fig. 4. First, the calibration geometry is moved into the OCT scanning region to scan the cross-sectional shape of the geometry (example scans are shown in Fig. 6). The next step is estimating the tip position and the associated error metric, which requires a computer vision algorithm, which is described in detail in Sec. 3.5.

The fitting error metric reflects the distance of the tool tip from the gathered cross-sections. If the error is above the predefined threshold, the robot pose must be adjusted by the operator or an algorithm. A simple greedy search is used to minimize the error because the metric is expected to converge. Once the fitting error for both scans is below the threshold, the tool tip position is determined. The determined point is moved to the center of the scan and to a set height (Z-position) in the B-scan. This configuration maximizes accuracy—because the geometry occupies most of the scan area—while still allowing the subsequent three-point calibration.

### 3.5 Cone Central Point Finding

If performed manually, the tip point can be located by adjusting the tool pose until two straight line segments are visible in the OCT cross-section. The intersection of these lines corresponds to the tip of the calibration geometry. When the tip moves out of the scan plane, the cross-sectional features quickly become curved, providing a clear visual cue and facilitating manual alignment.

**Fig. 5:**
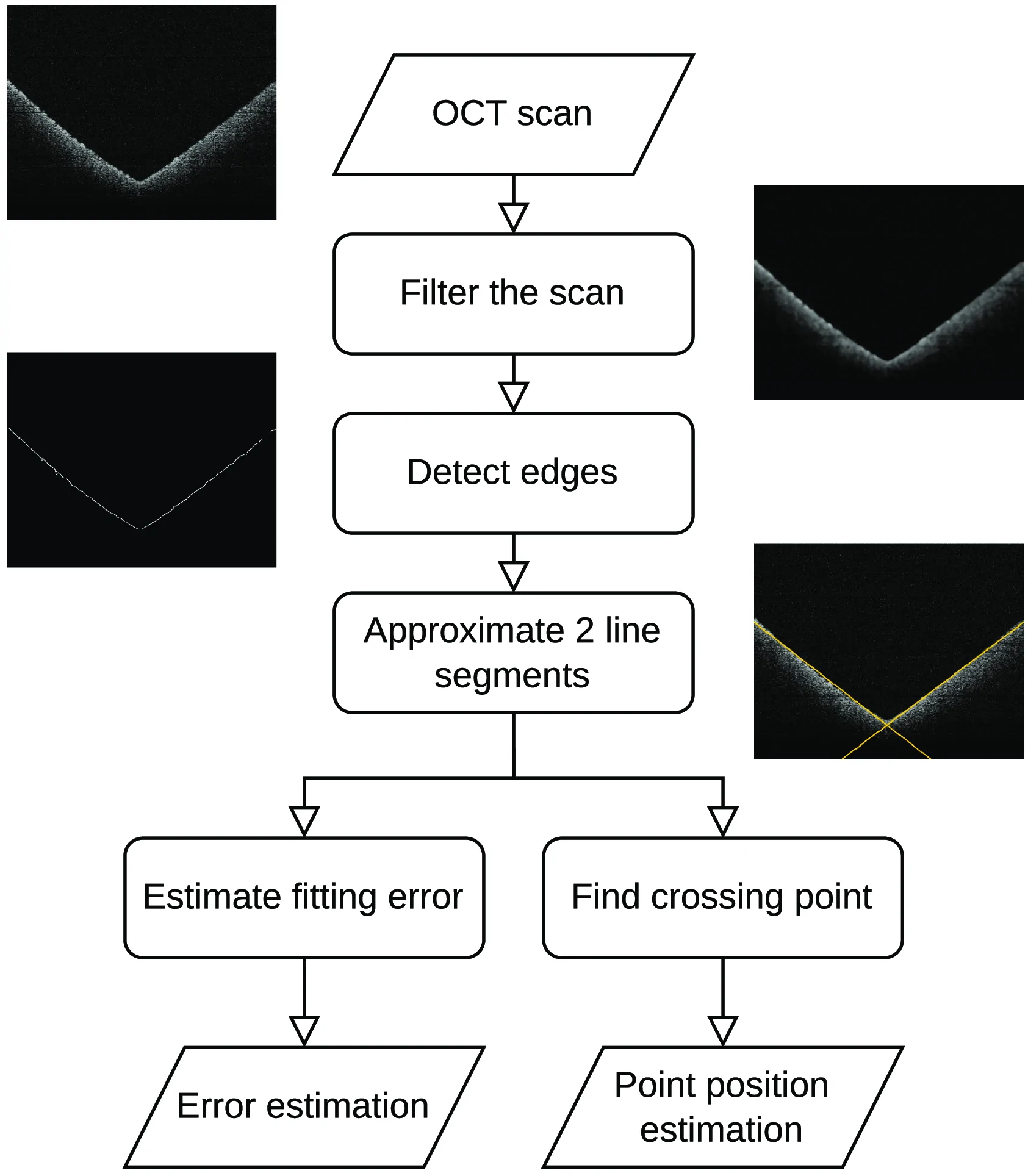
OCT-based algorithm for cone tip detection and error estimation.

The flowchart of the computer vision-based algorithm is shown in Fig. 5. The first processing stage is image denoising. This can be implemented using different filtering methods. An efficient way is to acquire multiple OCT B-scans at the same position and average them to improve the signal-to-noise ratio (SNR).^40^ OCT signal reconstruction is based on a fast Fourier transform (FFT). Zero-padding of the fringe signal before the FFT improves the precision of surface localization without impacting the system axial resolution. In our experiments, the best results were obtained by averaging 20 scans in a fixed position, followed by median and Gaussian filtering. Averaging improves SNR, median filtering suppresses salt-and-pepper noise while preserving edges, and the Gaussian blur reduces small edge irregularities before edge detection, resulting in more stable, continuous edge contours.

Once the image is adequately filtered, the edge detector can be applied to estimate the tool surface (we use the Canny detector as a representative choice). Because edge detectors are also sensitive to residual noise and intensity discontinuities, the previously described pre-filtering is essential. Additional preprocessing may be used to enforce the OCT-specific constraint that at most one surface point per A-scan column (i.e., per image column) is present.

Next, the extracted edge points are approximated by two straight line segments. Standard methods, such as fast line detector,^42^ can be used; however, they tend to fragment highly curved surfaces into multiple short segments. We obtained the most robust results using an $R^{2}$-based fitting strategy, which approximates any shape with a line and estimates the fit quality.

The $R^{2}$ method can estimate triangular shapes after splitting the image into two parts, each approximating one side of the shape. This approximation can be quickly applied to all X values, so no *a priori* knowledge is required. During the fitting step, each edge point is assigned to the closest line, and the best-fitting pair of lines is chosen. The positioning error (sum of $R^{2}$ values) of the best pair is intended to provide feedback on the distance between the cone tip and the OCT scanning plane. The cone-tip position is then estimated from a linear approximation of the cross-section defined by two fitted segments. This approach enables sub-pixel localization accuracy while remaining robust to minor imperfections in the calibration geometry.

**Fig. 6:**
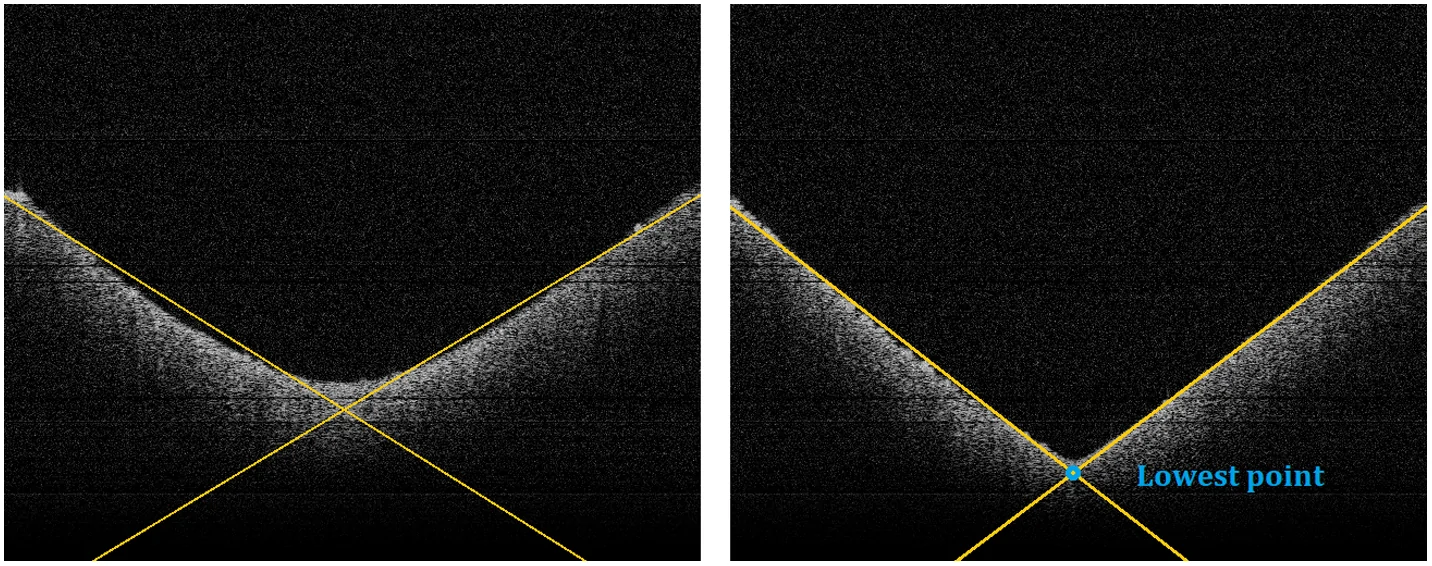
OCT result of the tool not being co-centric and after tuning. Yellow lines represent line approximation, and the blue point shows the estimated lowest point of the cone for the given image. Image on the left has high deviation value—gathered 1 mm off center.

Example results of the cone-tip detection and the associated fitting quality are shown in Fig. 6. On a standard PC-class computer, the calculations described above are completed under 1 s.

## 4 Results

Because the experimental OCT setup provides very high accuracy, the calibration error of the coordinate system can be quantified reliably only using the system’s own measurements. This analysis aims to perform the calibration over a short distance and then evaluate the resulting accuracy across the full OCT measurement, thereby estimating the error in single-point localization.

For this purpose, a 3D-printed calibration tool was fabricated using stereolithography, with the geometry shown in Fig. 3(a). The tool features a cone with a depth of 3 mm and a radius equal to 3 mm, corresponding to an included angle of 90 deg. The robot arm used in the experiment was the Mecademics Meca500. According to the manufacturer’s documentation, the position repeatability is 5  μm, and the path accuracy is 100  μm.

### 4.1 Error Estimation Method

To establish the error estimation method, we first analyze the impact of (small) position estimation errors on the calibration. The transformation is defined by three points: $P_{1}$ and $P_{2}$ define the X axis (and the origin), whereas $P_{3}$ defines only the direction of the Y and Z axes around X axis. Thus, by measuring positions along the X axis and defining a single-point positioning error value as the distance from the line:

$$e=\sqrt{y^{2}+z^{2}},$$ (7)

the errors introduced by imperfect $P_{3}$ placement are removed.

**Fig. 7:**
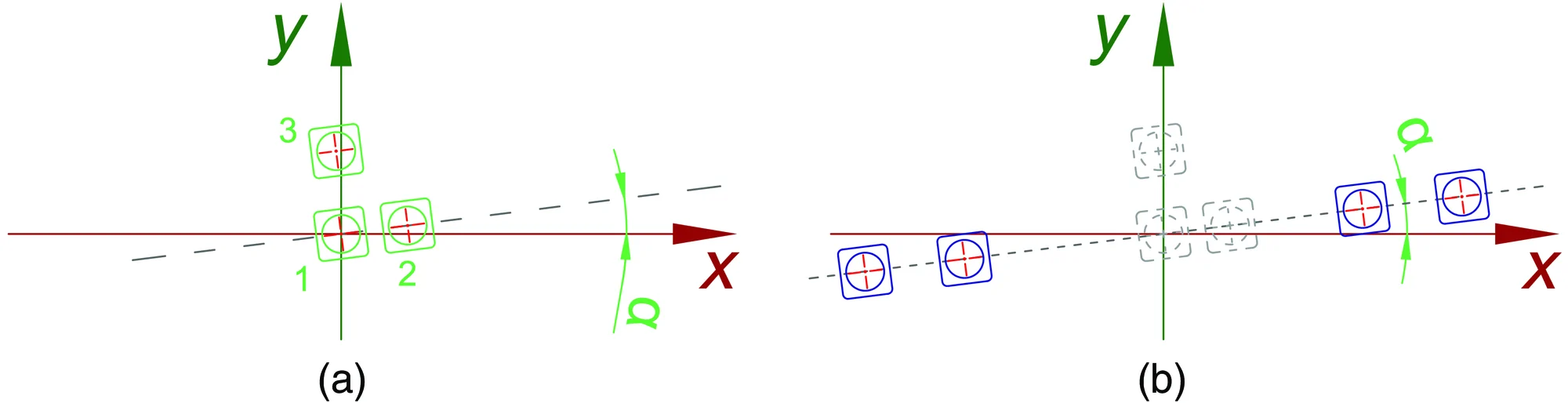
Visualization of the error estimation method used. Panel (a) presents primary calibration over short distance, whereas panel (b) presents error measurement over longer distances, which linearly scales the error up.

$P_{1}$ and $P_{2}$ define a line, which, considering nonperfect calibration, can be described similarly to a straight line equation in a 2D plane (y=ax+b) as a function:

e=ax+b=tan(α)·x+b, (8)

where α is the angular error X-axis alignment, and a,b are equation constants. α error results from linear errors of $P_{1}$ and $P_{2}$ estimation divided by the distance between them during the calibration phase. To increase the α-error values (and thus improve the accuracy of consecutive error estimation), the coordinate systems are first calibrated with the tool tip located at a short distance r from the origin of the OCT coordinate system [Fig. 7(a)].

The goal of the following error estimation is to define the parameters of Eq. (8) as accurately as possible. For that reason, multiple measurements are taken along the X axis over a possibly long range using a robot arm [Fig. 7(b)]—the higher the absolute X value, the larger the e value, and thus, the higher the relative accuracy. At each set position, the OCT system acquires a B-scan, re-estimates the cone-tip position, and adjusts the OCT scan pattern position until the best available fit is found. This enables the estimation of error by comparing the final position with the expected one. Such measurements allow us to estimate the error function for each performed calibration. By performing measurements for positive and negative X values, and averaging them, the influence of the offset b can be neglected as this value is expected to converge to zero with the increasing number of samples. The b constant is dropped, and the α error is calculated as α=atan(e/x). Using α instead of direct absolute distance comparisons reduces sensitivity to imperfect length reconstruction (scale) in OCT and isolates the dominant localization error, which does not result from the calibration method.

Finally, the statistical analysis is performed on the α values. The error values are nonnegative and should follow a half-normal distribution starting from e=0. The proposed alpha error margin will be defined as three standard deviations,

$$\Delta \alpha =3\cdot STD_{\alpha },$$ (9)

where $STD_{\alpha }=\sigma \cdot (1-\frac{2}{\pi })$ and Δα is the final confidence interval. To define the final linear positioning confidence interval, the Δα is applied to the length of the distance used for calibration r:

$$e_{\mathrm{pos}}=\frac{\tan(\Delta \alpha )\cdot r}{\sqrt{2}}.$$ (10)

The division by $\sqrt{2}$ accounts for uncertainty propagation because the estimated error results from two measurement errors, $P_{1}$ and $P_{2}$.^43^

Error estimation using line equations and angular inaccuracy instead of Euclidean was used for two reasons. First, it allows us to minimize the OCT system galvanometric scanners’ nonlinearity/errors. Computing the residuals directly from such positions would incorporate this OCT-intrinsic distortion into the calibration error estimate, conflating two distinct sources of inaccuracy. In our intended workflow, the robot-to-OCT coordinate calibration is performed first and subsequently used to drive a full OCT distortion characterization. Calibrating the OCT distortion before the error estimation would reduce the error, but residual distortion will still be measurable.

Second, the dominant source of error in the three-point coordinate-frame estimation is the inaccuracy in the recovered rotation matrix, which manifests as an angular misalignment of the calibrated axes. This angular error is nearly invisible in a single-point residual measured close to the calibration origin but grows linearly with distance. By calibrating at a short distance and measuring deviations at further positions from the origin and fitting the angular error coefficient of Eq. (8), we obtain an error metric that characterizes the transformation accuracy across the intended working volume rather than only at the calibration poses themselves. This is a more meaningful figure of merit for the surgical application, where the robot tip may operate anywhere within the OCT field of view.”

It is important to consider that the proposed error estimation method uses self-referential validation. This means that the result is lower-bound accuracy estimate or a precise estimation of the method’s consistency.

### 4.2 Final Positional Repeatability

**Fig. 8:**
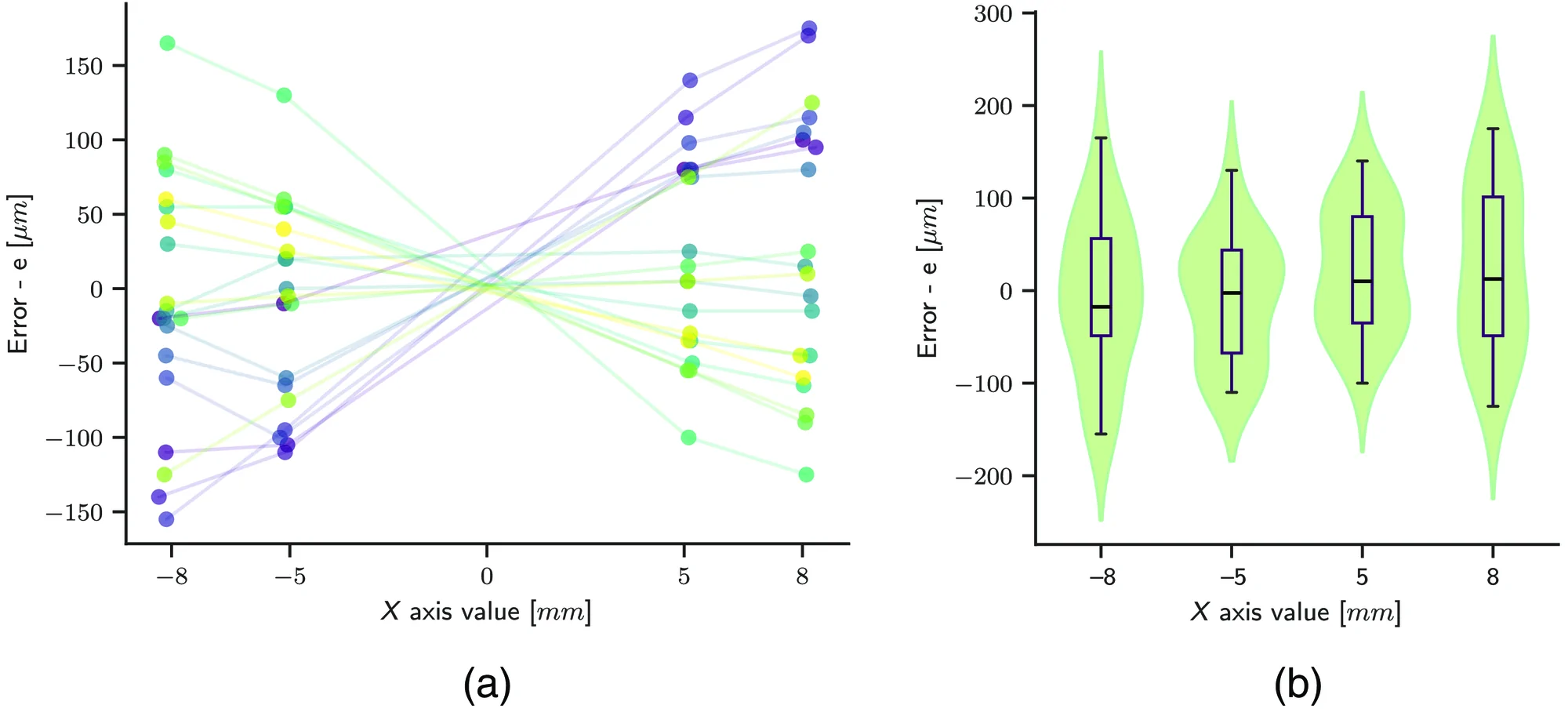
Chart presenting positioning errors measured 5 and 8 mm away from the coordinate system origin. For the purpose of interpretability, the error value was multiplied by the sign of y axis error. Panel (a) presents measurements grouped in color-coded fours per calibration. Panel (b) presents the distributions of the same measurements in groups per x axis.

The calibration error estimation was repeated 20 times. This included the calibration procedure and error estimation for displacements of 5 and 8 mm from the initial position in both directions (+X and −X). This resulted in 80 measurements (Fig. 8) and averaged 40 samples for the statistical analysis.

**Fig. 9:**
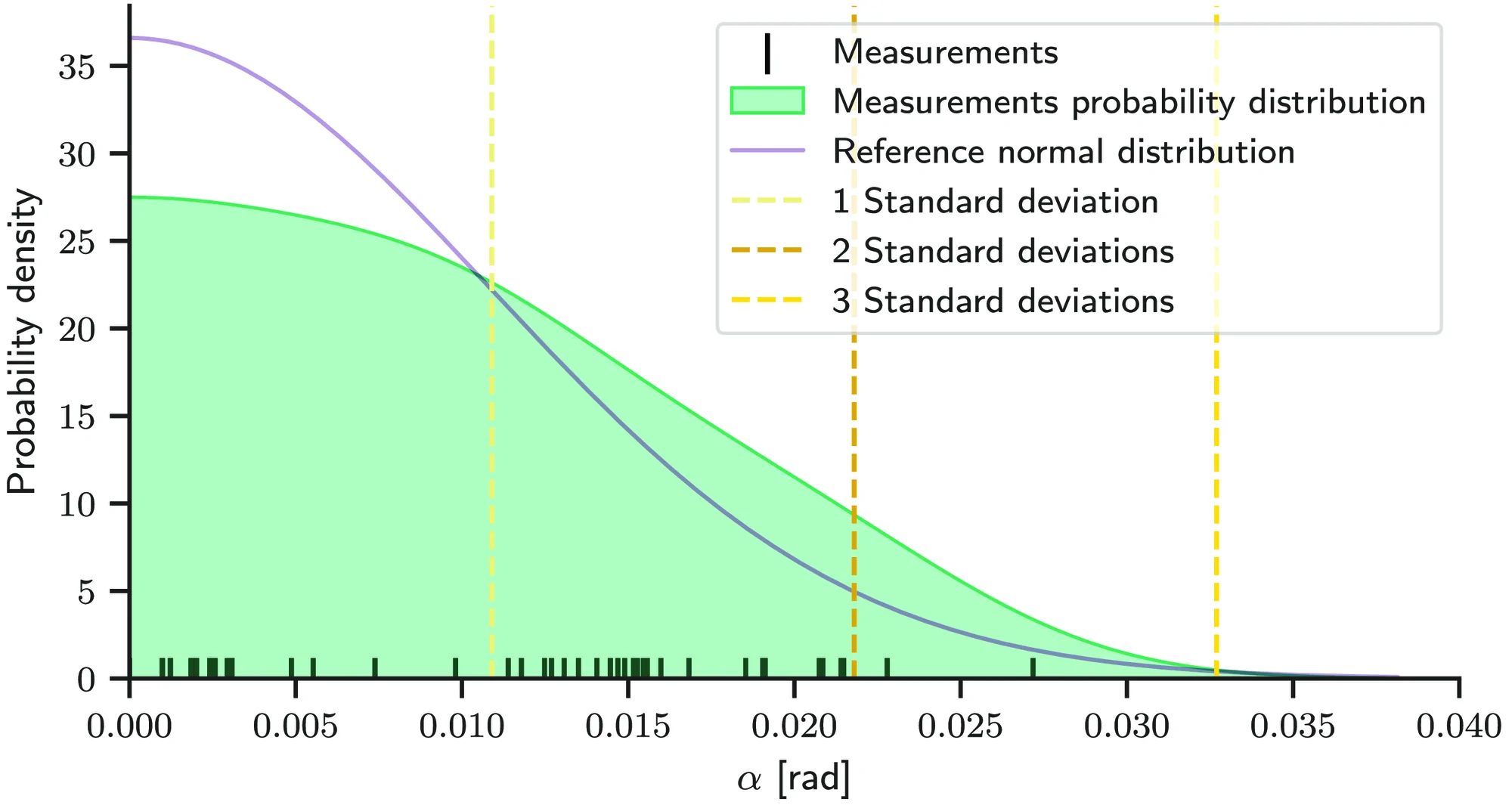
Probability density of the measured values and the resulting standard deviation ranges.

The measurements in Fig. 8(a) reflect the linear nature of the error introduced by imperfect calibration, whereas Fig. 7(b) presents the statistical parameters of the measurements. The biggest values were measured around x=8, reaching maximum of 175  μm. Measurements around +5 and −5  mm reached errors of up to 145  μm. For reference, a 26-gauge needle, classified as fine medical needle used for small-volume injections, has an approximate outer diameter of 450  μm, which is almost three times higher than the highest measured error for calibration over short r=1.5  mm range. The experiment’s half-normal distribution of angular repeatability is shown in Fig. 9. For reference, a perfect normal distribution is overlayed, and the ranges of 1 to 3 standard deviations are calculated for the gathered measurements. The presented measured distribution confirms the normal distribution hypothesis. The standard deviation was estimated as $\sigma_{\alpha }=0.01091\;\mathrm{rad}$. Therefore, a conservative angular confidence interval was

$$\Delta \alpha =3\sigma_{\alpha }\approx 0.0327\;\mathrm{rad}.$$ (11)

The resulting internal consistency of single cone position estimation can be calculated using Eq. (10):

$$e_{\mathrm{pos}}=\frac{\tan(0.0327)\cdot 1.5}{\sqrt{2}}\approx 34.7\;\mu m.$$

At this level, a substantial fraction of the measured error may be attributed to the robot’s inaccuracy rather than OCT localization alone. According to the manufacturer, the robot should reach each given position within 5  μm error margin, which translates to up to 14% of the $e_{\mathrm{pos}}$.

For comparison, a resolution-only calibration^22^ reported a 3.4% error at 270  pix/mm, which corresponds to ∼34  μm per 1 mm. With the method proposed here, the uncertainty of a two-point measurement over an OCT range L=16  mm can be approximated as:

$$\frac{\sqrt{2}\cdot e_{\mathrm{pos}}}{X_{\text{range}}}=\frac{\sqrt{2}\cdot 34.7\;\mu m}{16\;\mathrm{mm}}=3.11\;\frac{\mu m}{\mathrm{mm}}\approx 0.31\%,$$

leading to a visible improvement in the calibration accuracy. Even over r=1.5  mm calibration performed for this analysis, the inaccuracy stays lower (at ∼3.3%).

### 4.3 Calibration Time

The complete calibration procedure consists of three timing components. Robot motion between calibration poses takes typically around 1 s and the position adjustment takes around 300 ms for the Meca500 robot arm operating at moderate joint speed. OCT data acquisition requires ∼5  ms per frame at 200 Hz; with the 20-scan averaging used in our experiments, the total acquisition time per position is ∼100  ms. Image processing and cone-tip localization on a standard PC-class computer takes under 1 s as described above (740 ms on average). This means that every position adjustment consumes around 1.2 s when performed sequentially. If higher calibration accuracy is required, more sequences are typically performed. The end-to-end duration for a full three-point calibration presented in this article required around 7 to 9 adjustments for P1 and P2, and around 4 to 5 adjustments for P3, resulting in average 28 s calibration in automated mode. Direct comparison with a similar solution by Zhou et al.^18^ is not possible because calibration time was not reported; however, the 15-step manual procedure suggests a higher time burden for the operator.

It is important to mention that the most time-consuming component—the OCT image analysis can be significantly accelerated through multithreaded implementation in high-performance language like C++, or by offloading computation to a GPU.

## 5 Discussion

The described algorithms enable both manual and automatic calibration of coordinate systems between the robot and the OCT system. Using a conical shape reduced the influence of the noise in the OCT system and manufacturing inaccuracies. This resulted in a high final calibration accuracy, even with suboptimal geometries.

An additional benefit of the whole system is the ease of manufacturing the required geometry, using both additive and classical methods (for both convex and concave patterns). Existing tools can easily be modified to enable calibration.

A limitation of the primary error estimation method is that it uses the OCT system both for calibration and for measurement of the resulting error, making any systematic bias in the OCT measurements invisible to the evaluation. The reported 34.7  μm figure should therefore be interpreted as a lower bound on total system error rather than as an absolute accuracy claim.

**Fig. 10:**
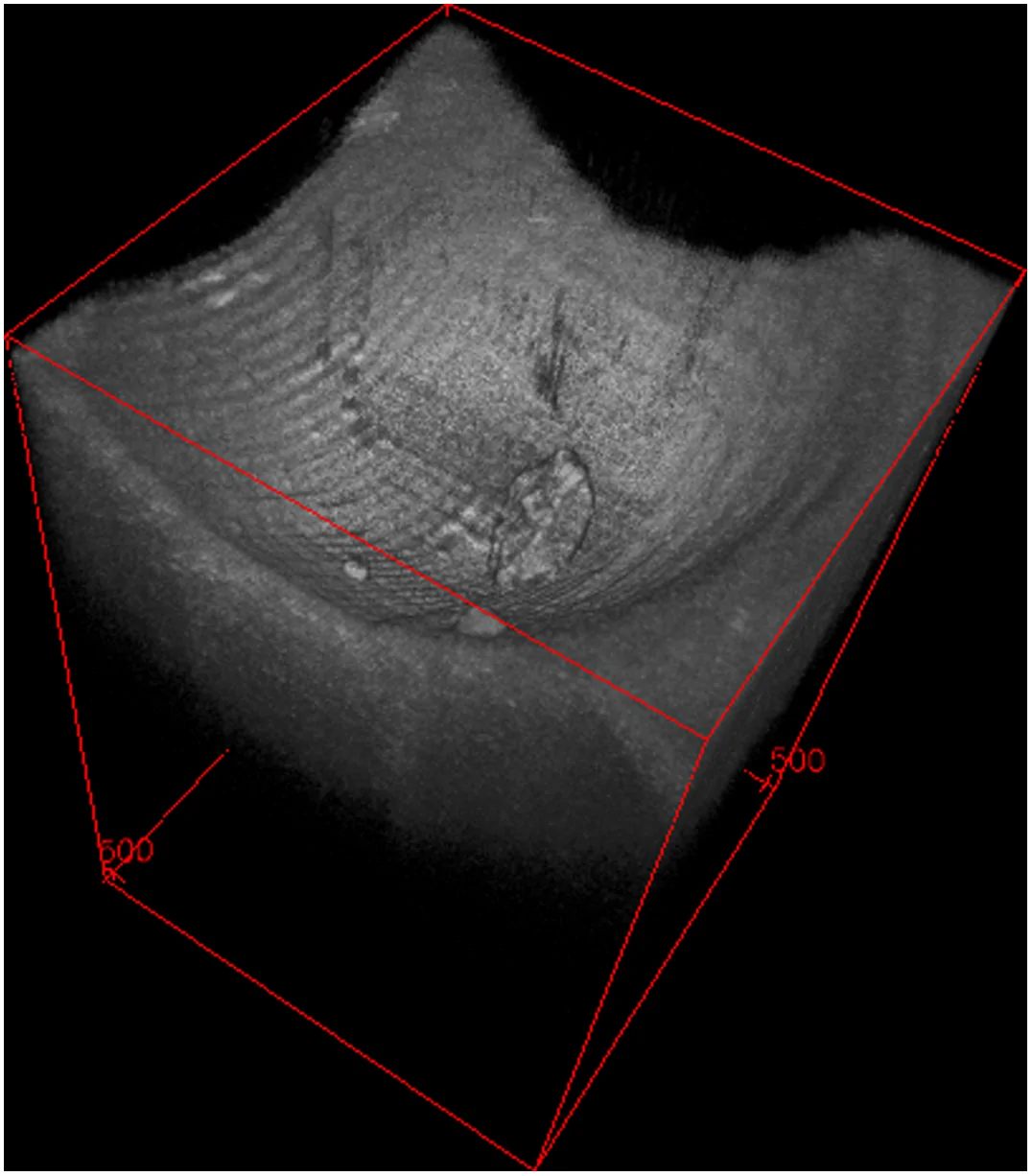
High-resolution 3D OCT scan presenting artifacts and the geometry of the cone used in the calibration resulting from the stereolithography manufacturing process.

On the other hand, a practical factor that limited measured calibration accuracy is the nonideal geometry of the calibration tool. In addition to robot positioning uncertainty, deviations from the nominal shape of the 3D-printed cone introduce systematic error into the OCT-based tip estimation. To assess this effect, the calibration geometry was additionally examined using high-resolution optical coherence microscopy, used in earlier works,^44^^,^^45^ which revealed local surface irregularities and manufacturing artifacts (Fig. 10). The presented 3D scan spans a space of 0.7×0.7×0.8  mm acquired with a pixel density of 512×512×2048  pixels for X-, Y-, and Z-axes, respectively.

These imperfections can affect the apparent cross-sectional profile of the cone and, consequently, the fitted line segments used for tip localization. Nevertheless, the proposed method remains relatively robust to such defects because it does not rely on detecting a perfectly manufactured apex; instead, it estimates the cone tip from the overall side-wall geometry. This makes the calibration less sensitive to moderate fabrication errors and supports the practical use of low-cost, easily manufacturable tools.

The accuracy of the calibration could be further improved by applying optimization methods typically used in the eye-to-hand calibration literature. This requires performing multiple measurements per each measurement point/axis at the cost of a longer calibration time.

Future work will focus on further improving both the transferability and the robustness of the calibration procedure. In particular, once the robot-to-OCT coordinate transformation has been established, the exact pose of the cone relative to the robot wrist should also be calibrated, which would be especially important for reproducible deployment across multiple devices or replacement tools. Additional improvements may be achieved by introducing more advanced filtering and image analysis methods to increase localization stability under lower signal-to-noise conditions. Finally, the convergence of the automatic alignment procedure could be improved by extending the cost function, for example, by incorporating the deviation of the angle between the fitted segments from the nominal cone geometry, particularly when the scan intersects the cone near its boundary.

Last, as stated in the introduction, this calibration aims to deliver information about the additional reference frames and does not universally translate to tool tracking. It can be used for tool tracking or additional data acquisition:

• In an open-loop control scheme:
  1. Using a simple approach where the robot-attached tool tip position is directly transformed into the OCT coordinate system, assuming negligible tool deflection and minor light refraction inside the tissue. This is valid for some anterior surgeries.
  2. Using a more complex approach that leverages structural information and the refractive indices of the eye components to model light refraction and scan geometry. It is important to note that the robot’s reference frame is not lost when entering the eye (e.g., during posterior surgeries). Instead, tracking errors occur because the OCT maps optical geometry, which differs from true physical geometry due to the refractive index of the ocular media and the optical beam scan geometry. Knowing these indices and the scan geometry enables the system to accurately translate the optical estimation into the true tool tip position, assuming minor ocular globe shifts, or crystalline lens accommodation changes. Should these become important, real-time anterior segment OCT scan to update the posterior scan geometry will be required for accurate tool tracking.
• In a closed-loop control scheme:
  3. Providing an expected pose for iterative OCT tracking systems. Using the robot’s kinematics for rough primary estimations, the OCT system can scan the space around the expected physical position to find the actual tool tip (in a narrow volume around the expected position). This provides a continuously updated offset for the next iteration, grounded in the knowledge of the robot’s physical movement, and can account for mechanical tool deflection.
  4. It can be used to increase the amount of information about tissue biomechanical and optical characteristics, when used in combination with additional external measurements (tool tracking from surgical microscope image analysis/real-time 3D OCT segmentation; or a force and torque feedback), as forces or refractions in the medium can be modeled based on the difference between expected and measured tool position.

In addition, establishing a highly precise robot-to-OCT transformation enables OCT system calibration. Galvanometric OCT scanners are voltage-driven. However, the input voltage linearity with the lateral scan distance is not always guaranteed due to optical and fan-distortion.^46^^,^^47^ Automation of cone-tip tracking enables scanning the OCT’s reachable area and creation of a high-resolution lookup tables for pose corrections (equivalent to an internal matrix for cameras).

A complete surgical validation—including an *ex vivo* eye or tissue-equivalent phantom with realistic refractive boundaries and a flexible surgical tool—is required before the method can be considered ready for tool tracking in intraoperative deployment. In future work, we plan to extend the applicability of the calibration to more realistic corneal surgery scenarios by introducing correction factors to the calibration based on real-time segmented corneal topography,^48^ and correction of the induced refraction and optical path length change.^49^ Similarly, additional transformations shall be applied to render the calibration relevant for posterior segment microsurgeries.^50^^,^^51^

## 6 Conclusion

We presented a calibration method for establishing the spatial transformation between a robot and an iOCT system for image-guided robotic-assisted microsurgical applications. The method combines a simple conical calibration tool, rapid 2D OCT cross-sectional imaging, cone-tip estimation from fitted line segments, and a vector-based coordinate-frame reconstruction procedure. This combination enables both manual and automated calibration without requiring volumetric OCT acquisition or complex fiducial geometries.

The experimental results show that the approach provides repeatable, accurate registration across the OCT measurement range while remaining robust to OCT noise, minor cone-manufacturing defects, and practical limitations of the robotic platform. Because the method relies on a geometry that is easy to manufacture and can be integrated into an existing tool holder, it is well suited for routine calibration in robotic OCT-guided surgery. Beyond robot-to-OCT registration, the same framework could be extended to map scanner distortions across the field of view and support lookup-table-based OCT correction. Future work should focus on refining the image-processing pipeline, improving the optimization cost function near boundary cases, and calibrating the cone’s exact pose relative to the robot wrist for transferable multidevice deployment.

## Data Availability

The algorithm Python implementation with an example scan is publicly shared via Zenodo (under https://doi.org/10.5281/zenodo.19039516) or GitHub (https://github.com/kgromada-ICHF/OCT-Calibration-Library).^52^ More calibration scans are available from the corresponding author upon request.
